# Predictive Clinical Indicators of Biochemical Progression in Advanced Prostate Cancer Patients Receiving Leuplin Depot as Androgen Deprivation Therapy

**DOI:** 10.1371/journal.pone.0105091

**Published:** 2014-08-14

**Authors:** Chien-Hua Chen, Ju-Ton Hsieh, Kuo-How Huang, Yeong-Shiau Pu, Hong-Chiang Chang

**Affiliations:** 1 Department of Urology, En Chu Kong Hospital, Taipei, Taiwan; 2 Department of Urology, National Taiwan University Hospital and College of Medicine, National Taiwan University, Taipei, Taiwan; National Health Research Institutes, Taiwan

## Abstract

Therapeutic planning and counseling for advanced prostate cancer patients receiving androgen deprivation therapy (ADT) is complicated because the prognoses are highly variable. The purpose of this study is to identify predictive clinical indicators of biochemical progression (BCP). In this retrospective analysis, data from 107 newly diagnosed patients (from November 1995 to April 2008) with advanced prostate adenocarcinoma receiving Leuprorelin acetate depot were analyzed. Data was collected from the computerized registry of two collaborating medical centers in Taiwan. Cox regression and Kaplan-Meier analyses were used to evaluate the relationship between potential predictive parameters and BCP. Univariate analysis revealed that predictors of BCP included (1) initial serum prostate-specific antigen (PSA) (hazard ratio [HR], 1.00; 95% confidence interval [CI] 1.00–1.00); (2) log of initial PSA (HR, 1.35; 95% CI 1.17–1.56); (3) PSA density at diagnosis (HR, 1.00; 95% CI 1.00–1.01), and (4) pathological bone fracture (HR, 2.22; 95% CI 1.20–4.11). Age (HR, 0.94; 95% CI 0.91–0.98) and hemoglobin levels (HR, 0.86; 95% CI 0.76–0.97) were also associated with greater risk of BCP. After adjusting for age, pathologic fracture, and hemoglobin level, the initial PSA and PSA density were no longer significantly associated with BCP. However, age and hemoglobin levels continued to be associated with greater risk of BCP (*P*≤0.007). Using Kaplan-Meier analysis, patients with higher initial PSA concentration, pathological bone fracture, and low hemoglobin had a greater probability of BCP. Thus, low hemoglobin and age are predictive indicators of BCP and therefore early indicators of BCP despite ADT therapy.

## Introduction

Prostate cancer treatment is diverse, and treatment decisions are often based on patient age, degree of invasiveness, and patient preference. For example, clinicians typically recommend radical prostatectomy for younger patients with early-stage and localized tumors. Conversely, androgen deprivation therapy (ADT) is currently the preferred method of treatment for metastatic and androgen-responsive prostate cancer.

ADT alleviates bone pain, decreases prostate-specific antigen (PSA) levels, and prolongs survival, and is therefore an effective treatment for metastatic prostate cancer [1]. ADT can be achieved through surgical (i.e., bilateral orchiectomy) or chemical castration. The most common form of chemical castration consists of monthly subcutaneous injections of a controlled-released dosage of gonadotrophin releasing hormone (GnRH, also known as Luteinizing-hormone-releasing hormone [LHRH]), such as Leuprorelin acetate depot, or daily oral intake of anti-androgens, including the non-steroid, Flutamide and Bicalutamide, as well as the steroid, Cyproterone acetate.

Up to 90% of patients receiving LHRH analogues have decreased serum PSA concentrations within the normal range [2]. Although not a cure, ADT can prolong the survival time of patients [1], [3]; it can also improve 80% of bone pain in these patients [4]–[6]. Although most patients initially respond to ADT, they eventually progress to a hormone-refractory state after approximately 18–24 months, with a median survival time of 24–30 months [7]–[9]. However, it is not unusual for patients to have a longer survival time and a hormone-responsive period greater than 24 months [6]. Thus, the prognoses of patients receiving ADT are highly variable [10], [11], complicating therapeutic planning and counseling.

Given the variable efficacy of ADT and its associated risks [12], predictive clinical indicators of BCP would facilitate therapeutic planning and counseling by giving an earlier indication of ADT failure prior to BCP. This study has included metastatic prostate cancer patients receiving ADT to analyze the relevant clinical related risk factors for BCP and used regression analysis for the prognosis prediction. This analysis may identify patients at risk of BCP and guide clinicians and patients regarding the appropriateness of applying first-line LHRH for patients with high initial PSA.

## Materials and Methods

### Study design

This is a retrospective cohort study. The pharmacy computer databases of two collaborating medical centers were queried to identify advanced prostate cancer patients on Leuprorelin acetate depot. In accordance with the patients’ inclusion and exclusion criteria, we selected eligible patients, and carefully documented their basic history, relevant blood tests, time points of diagnosis and treatment, and PSA levels prior to treatment and during treatment. The endpoint is BCP in advanced prostate cancer patients undergoing ADT. The study was approved by the medical research ethics committee of the School of Public Health in the National Taiwan University. All patients who participated in the study signed informed consent forms indicating that they knew of the existence of the study, understood its purpose, and agreed to participate.

### Patient data collection

Between November 1995 and April 2008, 107 patients with invasive or metastatic prostate cancer were treated with Leuprorelin acetate depot in the National Taiwan University Hospital and Cathay General Hospital. Patients meeting any of the following criteria were included: on Leuprorelin acetate depot for more than six months; initial diagnosis of invasive or metastatic disease; adequate follow-up; Patients meeting any of the following criteria were excluded: on Leuprorelin acetate depot for less than six months; initial diagnosis without invasiveness or metastases; change to the LHRH analogue therapy due to late bone metastases; orchiectomy; adjuvant LHRH analogue treatment after radical prostatectomy; and simultaneous LHRH analogue treatment and radiation therapy. The 107 patients selected had invasive or metastatic stage cancer and were treated with Leuprorelin acetate depot.

Various data was collected from the patient records, included the following data: (1) age at time of diagnosis; (2) body mass index (BMI); (3) initial PSA (taken within a month of diagnosis; (4) prostate volume as determined by transrectal ultrasound during transrectal prostate biopsy or transurethral prostate resection; (5) PSA density (initial PSA/volume of prostate); (6) hemoglobin (measured within a month of therapy initiation); (7) Gleason score; (8) clinical stage as determined by a radiologist using CT or MRI; (9) lymph node metastases; (10) bone metastasis; (11) soft tissue metastasis (to tissue other than bone and lymph nodes); (12) pathologic fracture as determined by the spread of cancer to the bone at diagnosis or treatment; and (13) Charlson Comorbidity Index [13].

A Gleason score of 7 was assigned as the cutoff value for the prognosis of malignancy as previously reported [14]. Also, as previously determined, a Charlson index above 7 indicated poor prognosis, and an index less than or equal to 5 indicated good prognosis [13].

### Determination of BCP

BCP was defined as a PSA value of at least 0.4 ng/mL followed by another two consecutive rises in serum PSA from a initial nadir value acquired by androgen deprivation [15], [16]. A drop in serum PSA was observed in all patients upon initiation of ADT. In BCP patients, serum PSA rises were observed while receiving ADT.

### Statistical analysis

Continuous variables were presented as mean and standard deviation (mean ± SD). Categorical variables were presented as numbers and percents. Depending on the serum PSA at the time of diagnosis, patients were divided into high and low initial PSA groups. Two sample t-test and chi-square tests were used to compare the different initial PSA groups in continuous and categorical variables. Logistic regression was used to estimate odds ratios (OR) and 95% confidence intervals (95% CI) of BCP during the follow-up period. Cox proportional hazard models were performed to indicate the hazard ratios (HR) and 95% CI of each variable at BCP rates. Variables with significant level (*P*<0.05) in univariate analyses were included into the multivariate model. Kaplan-Meier curves with log-rank test were used to evaluate the BCP-free rates and the differences for individual variables between two groups. Results were considered significant if the two-sided *P*-value<0.05. The statistical analyses were performed by SAS 9.2 statistics software (SAS Inc., Cary, NC, USA).

## Results

### The baseline study variables at the time of diagnosis and their relationship to initial PSA levels

The initial PSA levels among the various study variables at the time of diagnosis are presented in Table 1. Among the 107 patients, the mean age at the time of diagnosis was 73.75 y, and the mean BMI was 24.02 kg/m^2^. Since initial PSA and PSA density were skewed, we used natural logarithm to arrive at an average value of 4.93±1.79 and 1.23±1.68, respectively.

**Table 1:** Study variables at the time of diagnosis and their relationship to initial PSA levels^a^.

	Total(N = 107)	PSA≤106 ng/mL(N = 53)	PSA>106 ng/mL(N = 54)	*P*-value
Age (y)	73.75±7.93	74.81±6.47	72.70±9.07	0.169
BMI (kg/m^2^)	24.02±3.24	24.24±2.94	23.83±3.50	0.517
Prostate volume (cm^3^)	46.12±32.83	39.33±21.06	52.78±40.37	0.033*
PSA density at diagnosis (ng/mL/cm^3^)	19.44±71.87	1.39±1.30	37.15±98.41	0.010*
Log of PSA density at diagnosis	1.23±1.68	−0.03±0.89	2.47±1.31	<0.001*
Hemoglobin level (g/dL)	12.43±2.12	13.06±1.87	11.85±2.18	0.005*
Pathological fracture	19 (17.76%)	9 (16.98%)	10 (18.52%)	0.835
Distant metastasis	95 (88.79%)	47 (88.68%)	48 (88.89%)	0.973
Bone metastasis	93 (86.92%)	45 (84.91%)	48 (88.89%)	0.541
Gleason score				0.284
≤7	57 (53.27%)	31 (58.49%)	26 (48.15%)	
8, 9, 10	50 (46.73%)	22 (41.51%)	28 (51.85%)	
Hypertension	41 (38.32%)	19 (35.85%)	22 (40.74%)	0.603
Diabetes mellitus	18 (16.82%)	10 (18.87%)	8 (14.81%)	0.575
Coronary artery disease	10 (9.35%)	3 (5.66%)	7 (12.96%)	0.194
Cerebrovascular incident	6 (5.61%)	4 (7.55%)	2 (3.70%)	0.388
Charlson Index				0.841
≤5	12 (11.65%)	6 (12.00%)	6 (11.32%)	
6	68 (66.02%)	33 (66.00%)	35 (66.04%)	
≥7	27 (25.23%)	14 (26.92%)	13 (23.64%)	

a The median of initial PSA was 106 ng/mL.

*indicates a significant difference between the PSA groups.

Patients were divided into two groups based on initial PSA levels. Patients with initial PSA levels above the median (106 ng/mL) were placed in the high PSA group, and patients with initial PSA levels below the median were placed in the low PSA group. The prostate volume of the high PSA group was significantly greater than of the low PSA group (52.78 vs. 39.33 cm^3^, *P* = 0.033). In addition, the PSA density of the high PSA group was significantly greater than of the low PSA group (37.15 vs. 1.39 ng/mL/cm^3^, *P* = 0.010); similar results were observed with the log of PSA density values (2.47 vs. −0.03 ng/mL/cm^3^, *P*<0.001). Furthermore, hemoglobin levels were significantly lower in the high PSA group as compared to the low PSA group (11.85 vs.13.06 g/dL, *P* = 0.005). There were no significant differences in the other variables, including pathological fracture, metastasis, Gleason score, heart disease, diabetes mellitus, and Charlson index, between the PSA groups (Table 1).

### BCP rates by initial PSA level

As shown in Table S1, BCP rates between the high and low initial PSA groups were 17 BCP cases in 427.73 person-months in the low initial PSA group, and 37 BCP cases in 666.45 person-months in the high initial PSA group. Thus, there were 39.74 and 55.52 cases per 1000 person-months in the low and high initial PSA groups, respectively.

The odds ratios (OR) of BCP occurring in the high initial PSA group over the low initial PSA group are presented in Table 2. After adjustment for various parameters, including age and BMI in model 1; prostate volume, Gleason score, bone metastasis, and pathologic bone fracture in model 2; and hemoglobin at diagnosis, baseline hypertension, diabetes mellitus, cerebrovascular incident, history of coronary artery disease, and Charlson index in model 3, the OR indicates a significantly higher rate of BCP in the high initial PSA group (*P*<0.001).

**Table 2:** BCP in high and low initial PSA groups.

	OR (95% CI)	*P*-value
Model 1	4.85 (2.04–11.52)	<0.001
Model 2	5.33 (2.20–12.88)	<0.001
Model 3	4.94 (1.93–12.67)	<0.001

For all models, PSA≥106 vs. PSA<106, and the reference group was PSA<106.

Model 1 was adjusted for age and BMI.

Model 2 was adjusted for the following additional confounding factors: prostate volume, Gleason score, bone metastasis, and pathologic bone fracture.

Model 3 was adjusted for hemoglobin at diagnosis, baseline hypertension, diabetes mellitus, cerebrovascular incident, history of coronary artery disease, and Charlson index.

OR: Odds ratio; CI: Confidence interval.

### Univariate Cox regression analyses for BCP

The relationship between clinicopathologic parameters and BCP are presented in Table 3 as univariate Cox regression analyses. The following characteristics were associated with significantly greater risk of BCP (*P*<0.05): initial PSA (HR = 1.00), log of initial PSA (HR = 1.35), PSA density at diagnosis (HR = 1.00), and pathologic fracture (HR = 2.22). In addition, age (HR = 0.94, *P*-value = 0.002) and hemoglobin levels (HR = 0.86, *P*-value = 0.014) were also significant predictors of BCP. Both of these results revealed that younger age and anemia had higher risk for BCP. The other parameters tested show no significant effect on BCP.

**Table 3:** Univariate Cox regression analysis for BCP.

	HR (95% CI)	*P*-value
Age (y)	0.94 (0.91–0.98)	0.002
BMI (kg/m^2^)	0.98 (0.90–1.06)	0.568
Initial PSA (ng/mL)	1.00 (1.00–1.00)	0.015
Log of initial PSA	1.35 (1.17–1.56)	<0.001
Prostate volume (cm^3^)	1.00 (0.99–1.01)	0.700
PSA density at diagnosis (ng/mL/cm^3^)	1.00 (1.00–1.01)	0.001
Hemoglobin level (g/dL)	0.86 (0.76–0.97)	0.014
Pathologic fracture		
Yes vs. No (Ref, No)	2.22 (1.20–4.11)	0.011
Distant metastasis		
Yes vs. No (Ref, No)	1.68 (0.67–4.21)	0.273
Bone metastasis		
Yes vs. No (Ref, No)	1.73 (0.74–4.06)	0.207
Hypertension		
Yes vs. No (Ref, No)	1.00 (0.57–1.73)	0.987
Diabetes mellitus		
Yes vs. No (Ref, No)	1.06 (0.52–2.18)	0.865
Coronary artery disease		
Yes vs. No (Ref, No)	0.76 (0.28–2.11)	0.600
Cerebrovascular incident		
Yes vs. No (Ref, No)	0.31 (0.04–2.22)	0.243
Charlson Index		
6 vs. ≤5 (Ref, ≤5)	1.92 (0.75–4.89)	0.173
≥7 vs. ≤5 (Ref, ≤5)	1.18 (0.41–3.41)	0.195
Gleason score		
>7 vs. ≤7 (Ref, ≤7)	1.61 (0.94–2.74)	0.084

PSA, prostate-specific antigen; BMI, body mass index; CI, confidence interval.

### Multivariate Cox regression analyses for BCP

As shown in Table 4, multivariate Cox regression was performed on the variables that showed a significant risk of BCP. Because PSA density was derived from initial PSA, the variables were placed into two separate models – the first included initial PSA (model 1) and the second included PSA density (model 2). Initial PSA, age, pathologic fracture, and hemoglobin level were included in Model 1. After adjusting for age, pathologic fracture, and hemoglobin level, the initial PSA was no longer significantly associated with BCP (HR = 1.00, *P* = 0.342). Model 2 included PSA density, age, bone fracture, and hemoglobin. In Model 2, PSA density also was no longer significantly associated with BCP (HR = 1.00, *P* = 0.112). Age and lower hemoglobin level) were associated with greater risk of BCP in both models (Age: Model 1: HR = 0.94, *P* = 0.005, Model 2: HR = 0.94, *P* = 0.007; hemoglobin: Model 1: HR = 0.82, *P* = 0.004, Model 2: HR = 0.83, *P* = 0.007).

**Table 4:** Multivariate Cox regression analyses for BCP.

	HR (95% CI)	*P*-value
Model 1		
Initial PSA (ng/mL)	1.00(1.00–1.00)	0.342
Age (y)	0.94(0.90–0.98)	0.005
Pathologic fracture (Ref, No)	1.74(0.87–3.45)	0.117
Hemoglobin level (g/dL)	0.82(0.71–0.94)	0.004
Model 2		
PSA density (ng/mL/cm^3^)	1.00(1.00–1.01)	0.112
Age (y)	0.94(0.91–0.98)	0.007
Pathologic fracture (Ref, No)	1.77(0.89–3.50)	0.103
Hemoglobin level (g/dL)	0.83(0.72–0.95)	0.007

CI, confidence interval.

### Kaplan-Meier curves showing BCP-free probability

**Figure 1:**
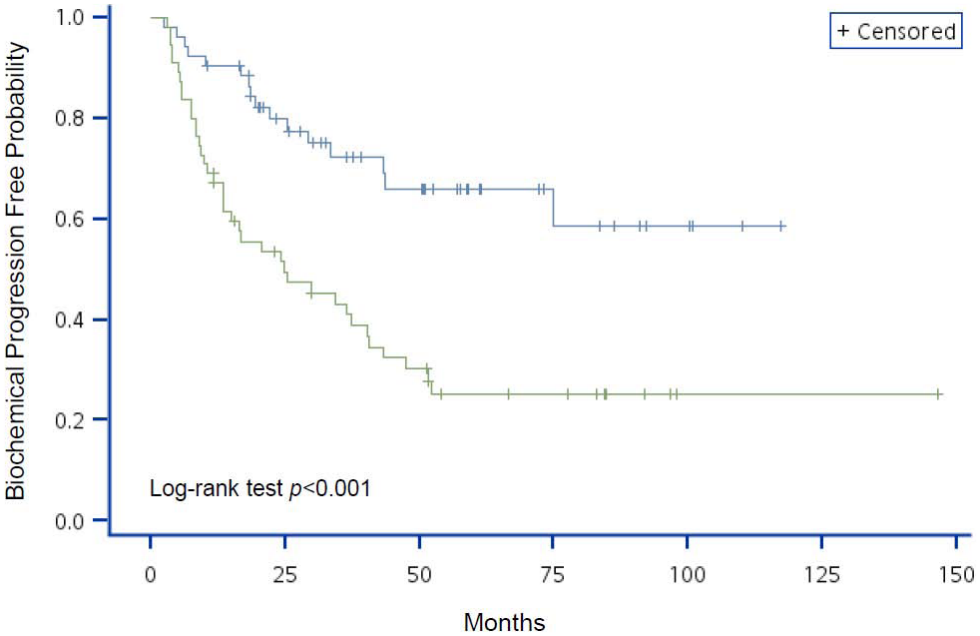
Kaplan-Meier analysis of BCP-free probability for advanced prostate cancer patients by risk group using initial PSA. The blue line represents the low initial PSA group (PSA<106 ng/mL) and the green line represents the high initial PSA group (PSA≥106 ng/mL). *P*-value of Log-rank test <0.001.

**Figure 2:**
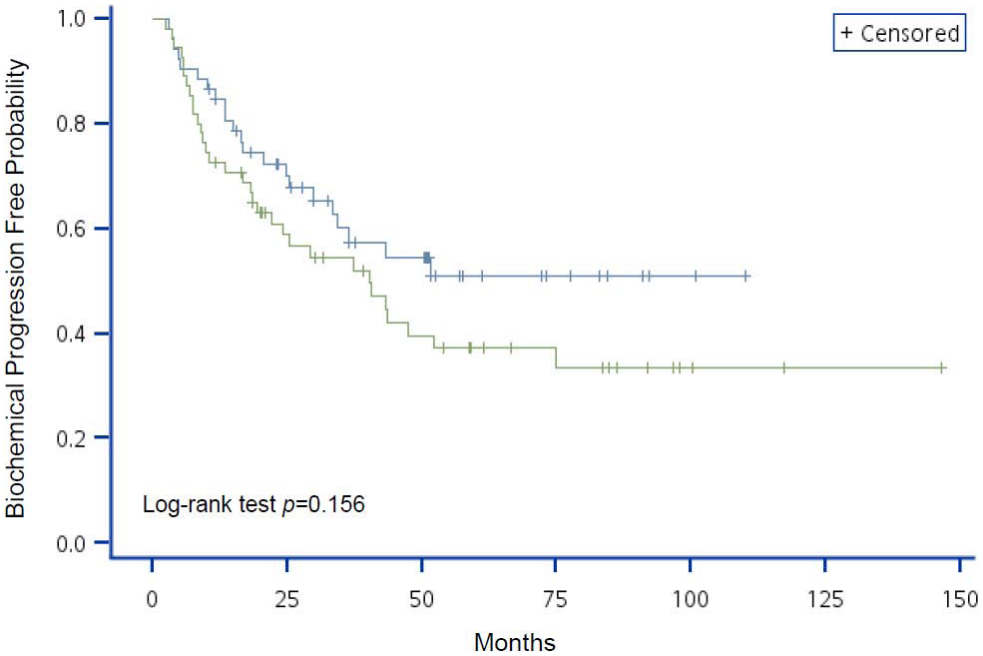
Kaplan-Meier analysis of BCP-free probability for advanced prostate cancer patients by risk group using age. The blue line represents age ≥74 y and the green line represents age <74 y. *P*-value of Log-rank test = 0.156.

**Figure 3:**
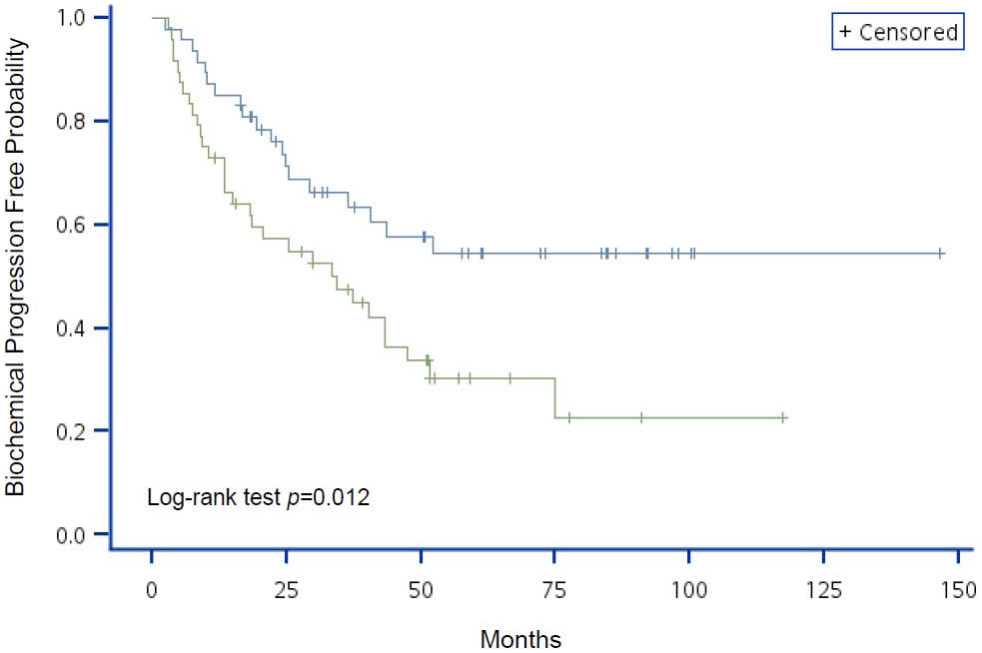
Kaplan-Meier analysis of BCP-free probability for advanced prostate cancer patients by risk group using hemoglobin levels. The blue line represents hemoglobin levels ≥12.7 g/dL and the green line represents hemoglobin levels <12.7 g/dL. *P-*value of Log-rank test = 0.012.

**Figure 4:**
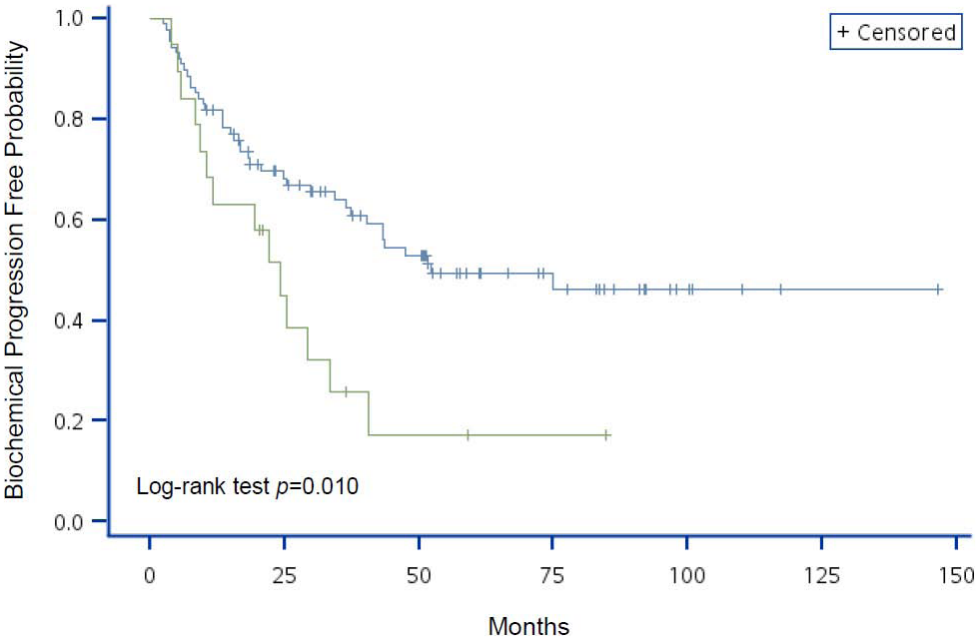
Kaplan-Meier analysis of BCP-free probability for advanced prostate cancer patients by risk group using pathologic fracture. The blue line represents patients without pathologic fracture and the green line represents patients with pathologic fracture. *P*-value of Log-rank test = 0.010.

The variables with significant HR on BCP in the univariate Cox regression analyses were used to generate Kaplan-Meier curves with log-rank tests (different to data presented in the tables where continuous variables were used where possible; variables were categorized in K–M curves). Figure 1 shows that patients with higher initial concentration of serum PSA had a greater probability of developing BCP (*P*<0.001). Although age was not a significant factor in BCP progression (*P* = 0.156; Figure 2), patients with lower hemoglobin levels or pathologic bone fracture had a higher probability of developing BCP (*P* = 0.012 and *P* = 0.010, respectively; Figures 3 and 4, respectively).

## Discussion

The efficacy of ADT is highly variable and known to induce both diabetes and cardiovascular disease [12]. Therefore, this study sought to identify predictive clinical indicators of BCP that will also be predictive of ADT efficacy. Two predictors of BCP were identified by univariate and multivariate analyses: (1) age and (2) hemoglobin levels. After adjusting for other covariates, these factors remained associated with BCP.

PSA is a diagnostic marker and biomarker of disease progression and efficacy of ADT [11], and its levels are reflective of the androgen, androstenedione, dehydroepiandrosterone sulfate (DHEA-S), and 3 alpha-androstanediol glucuronide levels during ADT [17]. In the present study, univariate analysis revealed that patients with initial PSA concentrations >106 ng/mL and higher PSA density at diagnosis had an increased risk for BCP. In the present study, serum PSA and therefore PSA density values had a skewed distribution. After the natural logarithm conversion, initial PSA levels remained a risk factor for BCP. This is in agreement with Huang et al. [18], who reported that initial PSA, PSA nadir ≥2 ng/mL, and shorter time to PSA nadir was associated with disease progression. In patients with bone scan negative prostate cancer, PSA nader ≤0.1 ng/mL and greater time to nadir (>24 months) had good prognoses [19]. In addition, Keto et al. [20] reported that PSA nadir predicted progression to castration-resistant prostate cancer in patients treated with ADT after radical prostatectomy.

The association of initial PSA and BCP observed in the present study is also consistent with previous reports of a correlation with serum PSA concentration and patient survival [21]–[25]. For example, in patients with advanced prostate cancer treated with ADT and radiation therapy (RT), initial response to ADT prior to RT was predictive of survival [24]. However, other studies did not find such a correlation [26]. For example, a rapid decrease in PSA levels was predictive of disease progression and poor survival in patients with localized disease and treated RT with and without ADT [27]. Furthermore, a shorter time to PSA nadir was predictive of shorter survival [27]–[31].

PSA density at diagnosis was also associated with increased risk of BCP by univariate analysis. This is in agreement with Radwan et al. [32] that reported that PSA density can be used as a predictor of BCP in prostate cancer patients after radical prostatectomy. For clinically localized prostate cancer, PSA density may be representative of the extent of tumor invasion, and can be used as predictor of prognosis before the treatment. Most of the patients in this study had invasive tumors that extended beyond the margin of the prostate; therefore, determining PSA density at diagnosis, which accounts for prostate gland volume, may best determine risk of BCP in these patients.

In addition to PSA and PSA density at diagnosis, pathological fracture also increased the risk of BCP by 2.22-fold in the present study. While this is the first study to find such a correlation, other studies in metastatic prostate cancer patients suggest that bone pain is a predictor for survival, increasing risk by 2.5-fold [21]. However, because prostate cancer patients are primarily older, bone pain may not be a result of bone metastasis, but due to degenerative arthritis or other muscle and joint problems. Therefore, we selected pathological fracture, which is not subjective as in bone pain.

In the present study, lower hemoglobin levels increased the risk of BCP by univariate and multivariate analyses. This is consistent with Cho et al. [33], who reported that elevated hemoglobin levels had the strongest protective effect in response to hormonal failure on relative factors affecting the survival of patients. In addition, Jørgensen et al. [34] concluded that hemoglobin levels higher than the 13.5 g/dL increased survival by 21% in patients with metastatic prostate cancer.

Many studies found that age increased risk of survival in patients with metastatic prostate cancer by 1.49- to 2.5-fold [21], [22], [24], [33]. However, several studies found no obvious correlation between age and survival [25], [34]. In the present study, age is also a risk factor of BCP; therefore, age may have a protective role with respect to BCP. The median age at diagnosis was 73.8 y, which is older than previous studies of European, American, and Scandinavian populations [8], [34], [35]. However, it was similar to another study of Taiwanese patients, whose median age was 75.7 y [21]. It is possible that recent advances in screening and diagnostic tools may detect these patients earlier. In addition, given the low incidence of prostate cancer in Taiwan (i.e., 21.94 cases per 100,000 [36]), PSA screening is not routine. Although it is possible that younger men may have more malignant and aggressive metastatic cancer, further prospective studies and clinical trials are necessary to understand the relationship between age and BCP.

The impact of performance status on survival is conflicting [22], [26], [33], [34], [37]. Although performance status was not assessed, the presence of comorbid conditions was evaluated using the Charlson index. However, no significant correlations were observed with respect to BCP. In addition, no correlations between Gleason score and BCP were observed. This is in contrast to previous studies that observed a correlation between high tumor grade and survival [21]–[23]. However, other studies found no such correlation [25], [32], [33]. Moreover, no tissue biomarker, including Ki67 and p16, has been reported to have conclusive predictive value for the outcome of prostate cancer treatment [38].

In a previous study, patients with BMI>27 kg/m^2^ had 2.5-fold greater risk with respect to survival [25]. However, no such correlation was observed between BMI and BCP. This might be due to the relatively low BMI of our patients as the average BMI was 24 kg/m^2^ with few have >27 kg/m^2^.

Most previous studies of prognostic indicators in prostate cancer use death as the end-point of the study. Furthermore, most include patients with localized prostate cancer receiving a definite treatment or those receiving chemotherapy for metastatic disease following failed ADT. However, our study used BCP as the surrogate end-point because the goal of this study was to identify factors to inform treatment decisions.

This relatively small size and retrospective nature of the study represents a study limitation. This is partially due to the relatively low incidence of prostate cancer in Taiwan as compared to Western countries [36]. Secondly, the present study did not assess alkaline phosphotase level, performance status, and bone pain, which are clinical factors related to the survival of prostate cancer patients [21,22,26,33,34,37,39]. Furthermore, this study had no information regarding patient survival.

## Conclusions

In this study, we have identified two independent predictive clinical indicators of BCP in Taiwanese patients with advanced prostate cancer: (1) age and (2) hemoglobin levels. Identification of patients at risk of BCP may inform treatment decisions with respect to the appropriateness of applying first-line LHRH. These findings need to be confirmed by further large-scale prospective studies.

## Supporting Information

Table S1 **BCP rates between high and low initial PSA groups.**
(DOC)
