# Supplementary material for: Predictive Clinical Indicators of Biochemical Progression in Advanced Prostate Cancer Patients Receiving Leuplin Depot as Androgen Deprivation Therapy
Source: PLoS One. 2014 Aug 14;9(8):e105091. doi: 10.1371/journal.pone.0105091 (PMC4133301; doi:10.1371/journal.pone.0105091)
Supplement: Table S1 — BCP rates between high and low initial PSA groups. (DOC) [file pone.0105091.s001.doc]

**Supplemental Table 1. BCP rates between high and low initial PSA groups.**

|  | PSA | |
| --- | --- | --- |
|  | ≤106 ng/mL | >106 ng/mL |
| Cases with PSA biochemical progression | 17 | 37 |
| Person-month^a^ | 427.73 | 666.45 |
| Rates (%)^b^ | 39.74 | 55.52 |

^a^Person-month means total months from cases during follow-up period.

^b^Rates are cases with BCP per thousand person-months.
